# White wines aged in barrels with controlled tannin potential exhibit correlated long-term oxidative stability in bottle

**DOI:** 10.1016/j.fochx.2024.101907

**Published:** 2024-10-16

**Authors:** Kevin Billet, Cécile Thibon, Marie Laure Badet, Nolwenn Wirgot, Laurence Noret, Maria Nikolantonaki, Regis D. Gougeon

**Affiliations:** a Université de Bourgogne, Institut Agro, INRAe, UMR PAM 1517, Institut Universitaire de la Vigne et du Vin – Jules Guyot, F-21000 Dijon, France; b Université de Bordeaux, Bordeaux INP, INRAE, UMR 1366 OENO, ISVV, F-33140 Villenave d'Ornon, France; c Œnologie By MLM, 57 Rue de la Paix, 33140 Villenave d'Ornon, France

**Keywords:** Chardonnay, Sauvignon blanc, Aging, metabolomics, DPPH, Liquid chromatography mass spectrometry, Gas chromatography mass spectrometry

## Abstract

Chardonnay and Sauvignon blanc wines aged in oak wood barrels with low and medium tannin potentials were discriminated for their abilities to resist against oxidation during bottle storage. The oak wood tannin potential was positively correlated to wines antioxidant capacity after 2 and 4 years of bottle aging. Untargeted molecular analysis revealed that the Sauvignon blanc metabolome was more affected by the tannin potential than the Chardonnay. Supervised statistical analysis highlighted the extensive oak wood contribution to the wine chemical fingerprints. Wines aged in barrel of medium tannin potential were associated with higher concentrations in antioxidant compounds such as dipeptides. Moreover, quantitative differences were observed between oak barrel derived volatile compounds. Sauvignon blanc volatile thiols appeared to decrease during bottle aging, regardless of the oak tannin potential. This study highlights the post bottling positive impact of oak wood barrel aging on wines oxidative stability, related to oak barrel tannin potential.

## Highlights

• Discrimination of bottled white wines according to the tannin potential of barrels they were aged in.
• Antioxidant capacity of Chardonnay and Sauvignon blanc is positively correlated to the tannin potential.
• Barrels with medium tannin potential lead to wines with higher concentrations in antioxidants.
• Chardonnay antioxidant metabolome evolution is less sensitive to the tannin potential.

## 1 Introduction

Wine chemical composition is resulting from complex contributions from vintage, grape variety, viticultural and winemaking practices. These factors greatly affect both wine primary and secondary metabolites related to the expression of varietal typicality and shelf-life (Lee et al., 2009; Roullier-Gall et al., 2014). Oak wood related metabolites are generally associated with wines sensory complexity and oxidative stability (Nikolantonaki et al., 2019). From the enological perspective, oak wood barrel is now understood as an active reactor that enable wood-wine gas and molecular exchanges (Carpena et al., 2020; Gougeon et al., 2009). Multiple factors are known to influence the gas exchange capacity among which the within barrel pressure drop, the headspace formation, the wood anatomy, its moisture as well as the barrel toasting (Del Alamo-Sanza & Nevares, 2018). During the barrel storage, a molecular dialogue occurs between the wine and the oak wood which leads to ellagitannins, quercotriterpenosides and volatile constituents extraction (Călugăr et al., 2020; Gougeon, Lucio, De Boel, et al., 2009; Marchal et al., 2015). The wine micro‑oxygenation triggers numerous chemical modifications, particularly of the ellagitannin pool (García-Estévez et al., 2017) even once the wine is bottled.

Oak wood ellagitannins are major substrates for oxidation and lead to colour change due to their polymerization (Glabasnia & Hofmann, 2007; Jeremic et al., 2020; Jourdes et al., 2011) but also contribute to wine's oxidative stability during aging (Alañón et al., 2011) depending on their physicochemical structure. Tannin potential and oak wood toasting influence white wine antioxidant capacity by preserving the molecular pool of the native antioxidant metabolome of wines (Nikolantonaki et al., 2019). As an easy and efficient method used to determine the antioxidant capacity of many beverages, the 2,2-Diphenyl-1-picrylhydrazyl (DPPH) is commonly used to assay wines oxidative status (Mitrevska et al., 2020; Romanet et al., 2021; Tekos et al., 2021). As a mixed-mode assay, the DPPH radical deactivation occurs through both hydrogen atom (radical quenching) and electron transfer (direct reduction) (Apak et al., 2016) and is easily monitored through spectrophotometry in the range 515–520 nm (Danilewicz, 2015; Sharma & Bhat, 2009). Conveniently, antioxidant capacity is expressed as the sample efficient concentration that enabled the reduction of 50 % (EC_50_) of the initial DPPH free radical amount. To overcome the lack of relationship between DPPH discoloration and the sample concentration, nowadays the EC_20_ appears to be a consensus for white wine solutions after optimization of the buffer solution (Romanet et al., 2019).

Recently, pairing metabolomics with wines chemical antioxidant capacity monitoring was proved as a promising tool for the study of white wines oxidative stability (Romanet et al., 2021). Wine targeted metabolomics enabled grape varieties discrimination through biomarkers identification when untargeted approaches contributed to considerable progress in the wine chemical elucidation (Roullier-Gall, Witting, et al., 2014). Metabolomics coupled to chemometric tools revealed wine unexpected fingerprints, associated with the geography of cooperage oak (Gougeon et al., 2009) or with centennial winemaking practices (Jeandet et al., 2015). However, studies dealing with wine oxidative stability during bottle aging in their majority monitored the evolution of potent volatile oxidation or varietal volatile molecular markers in relation with the dissolved oxygen levels and/or trace metal impact (Ugliano et al., 2020).

In order to gain control of how wines barrel aging conditions impacts its shelf-life, the present study proposes to assess the oak wood tannin potential influence on the volatile and non-volatile molecular signature and on the antioxidant capacity of barrel-aged white wines, after 2 and 4 years of bottle aging. Untargeted UPLC-Q-ToF-MS analysis, targeted GC–MS/MS and the global antioxidant capacity assay were conducted on Chardonnay (CHA) and Sauvignon blanc (SAU) wines aged on lees for 8 months in barrels with controlled low (LTP) or medium tannin potentials (MTP).

## 2 Materials and methods

### 2.1 Chemicals

Acetonitrile (C_2_H_3_N; 99.99 %) and the formic acid (CH_2_O_2_; 99 %) were LC-MS grade and came from Thermo Fisher Scientific (Courtaboeuf, France) while methanol (CH_4_O; 99.9 %) was obtained from Honeywell (Levallois Perret, France). Ultrapure water (18.2 MΩ.cm^−1^ resistivity) was provided by a Millipore Milli-Q water purification system (Merck Millipore, Molsheim, France). 2,2-Diphenyl-1-picrylhydrazyl (DPPH), the citric acid and the phosphate dibasic were purchased from Sigma-Aldrich (Saint Quentin Fallavier, France).

### 2.2 Wine samples and oak barrel characteristics

Chardonnay wines (CHA) from Burgundy region and Sauvignon blanc (SAU) wines from Bordeaux region, all from the 2016 vintage, were aged on lees in commercial french oak barrels with controlled tannin potential. Barrel tannin potential classification relied on the ellagitannin content quantitation based on near-infrared spectroscopy (NIRS, AOTF, Brimrose, USA). This device provides all together a rapid, high-resolution and reproducible spectral scanning. It ranks oak wood barrels in low tannin potential (LTP) or in medium tannin potential (MTP), which corresponds to LTP: 2000–4000 μg and MTP: 4001–6000 μg of ellagic acid equivalent per gram of dry wood, respectively. The barrel toasting was identical and resulted from heating staves one hour at 150 °C using a radiant heat with a heating accuracy of ±3 °C. After 8 months of barrel aging, all wines were filtered, bottled with a micro-agglomerated cork and stored until analysis at cellar temperature (14–16 °C). Wines were analyzed in biological replicates (CHA/2y: *n* = 6, CHA/4y: *n* = 10; SAU/2y: *n* = 4 SAU/4y: n = 6) at 2 (2y) and 4 (4y) years of bottle aging (Supplemental Table 1).

### 2.3 Determination of the 2,2-diphenyl-1-picrylhydrazyl (DPPH) antioxidant capacity

The wine ability to scavenge DPPH radical was assayed using the optimized protocol proposed by (Romanet et al., 2019). In brief, 27 mg of DPPH were dissolved in 1 L of citrate-phosphate:methanol buffer (0.3 M; 60:40, v:v, pH 3.6). Calibrations curves were made for each sample and resulted from the addition of increasing wine volume (0 μL to 100 μL) mixed with 3.9 mL of DPPH solution, under an oxygen-free atmosphere. Samples (*n* = 2) were incubated for four hours in the darkness. Then, the normalized absorbance (blanks: n = 6, 0.1 mL ethanol: water, 12:88, v:v, pH 3.2 in buffer) at 525 nm was measured using an UV-1800 Shimadzu UV–Vis spectrophotometer (Marne-la-Vallée, France). Results were expressed as the wine volume needed to reduce the initial DPPH absorbance by 20 % (EC_20_).

### 2.4 UPLC-Q-ToF-MS/MS analysis

Untargeted metabolomic analyses were conducted using a Dionex Ultimate 3000 UHPLC (Thermo-Fisher Scientific Inc., Waltham, MA USA) coupled to a maXis Plus UHR-ToF-MS (Bruker, Bremen, Germany) equipped with an electrospray ionization source (ESI) set in positive mode and controlled by the HyStar 5.1 software (Bruker Daltonik, Bremen, Germany). Sodium salt of formic acid (0.05 % formic acid, 1 % NaOH (0.1 M), isopropanol:water (1:1, v:v)) was directly injected into source to perform the external calibration of the mass spectrometer. This Na Formate solution was injected through a bypass system between 0.1 and 0.3 min to process to the internal calibration using the “enhanced quadratic” mode with an error lesser than 0.5 ppm. Samples were randomly injected with a volume set at 5 μL and the analytes separation used a reverse phase Acquity BEH C18 (1.7 μm, 100 × 2.1 mm) column (Waters, Guyancourt, France) with a flow rate of 0.4 mL.min^−1^ at 40 °C. Eluent A was composed of acetonitrile:water (95:5, v:v) and eluent B was made of acetonitrile both supplemented with 0.1 % of formic acid. The chromatographic separation consisted in an isocratic step from 0 to 1.10 min with 5 % (v:v) B, then B eluent increased through a linear gradient until 95 % (v:v) at 6.40 min maintained for 3.60 min and returned to starting condition in 0.20 min for 5 min of re-equilibration. Acquisitions were performed in positive ionization mode with the following parameters: nebulizer pressure of 3 bar, nitrogen gas flow and temperature respectively set at 10 L.min^−1^ and 200 °C), a 4.5 kV capillary voltage, end plate off set fixed at +500 V and a mass range between 50 and 1500 Da. Fragmentation (MS^2^) was realized at 8 Hz spectra rate using autoMS/MS function (20–50 eV).

### 2.5 GC–MS/MS analysis

Gas chromatography analysis protocol and system was adapted from Thibon et al. (2015). In brief, wine samples (20 mL) were spiked with 20 μL of internal standards: 6-sulfanylhexanol (6SH), 4-methoxy-2-methyl-2-sulfanylbutan (MMSB), ethyl maltol (EM) and 3-octanol respectively made using 30 μmol.L^−1^ for 6SH and MMSB and 100 μmol.L^−1^ of EM and 3-octanol. Samples were percolated through a conditioned SPE column (HR-X, 500 mg 6 mL, Macherey Nagel, France). Then, SPE columns were rinsed twice with 2 mL of hydro-alcoholic solution (10 %) before eluting by 3 mL of pentane/dichloromethane (50/50; *v*/v) followed by 3 mL of dichloromethane/methanol (95/5; v/v). The organic phases obtained were blended, dried over anhydrous sodium sulfate, and concentrated to 100 μL under a nitrogen stream.

The chromatographic system was made of a Trace GC Ultra gas chromatograph (Thermo Electron SAS, Courtaboeuf, France) coupled to a triple quadrupole mass spectrometer TSQ Quantum XLS using electronic impact and equipped with a Triplus RSH auto-sampler. The separation was achieved by a polar Optima WAXplus column (polyethylene glycol, 60 m; 0.25 mm; 0.25 μm) from Macherey Nagel (Hoerdt, France). Helium was used as carrier gas with a flow rate set at 1 mL.min^−1^; He was provided from Messer France SAS (Suresnes, France; 6.0 grade). One microliter of sample was injected into a split/splitless programmable-temperature injector (closure time: 1 min, split flow 30 mL.min^−1^). The gradient started with a temperature set at 180 °C until 0.01 min, raised at 230 °C at 14.5 °C.min^−1^, the temperature was maintained for 1 min and then raised until 250 °C at 14.5 °C.min^−1^, maintained for 10 min. The initial oven temperature was set at 45 °C, kept for 1 min and reached 250 °C at 4 °C.min^−1^, raised to 260 °C at 20 °C.min^−1^ finally maintained for 10 min. The MS transfer line temperature was set at 260 °C.

### 2.6 Data mining

The sample injection plan started by a column conditioning of 90 min followed by the injection of five blanks to ensure the system equilibration. Then, five QCs (pool of samples) injection occurred, followed by one new QC injection each 10 randomly selected samples. The sample plan ended with five more QCs injection to control the reproducibility and the analytical variability. Data preprocessing was performed using MetaboScape MS software (Bruker, Bremen, Germany), a minimum of 20 % of features was retained for extraction and presence over analyses. The processing method used T-ReX 3D algorithm to align retention times, do the deisotoping and feature (intensity threshold set at 1000 AUC with a minimal peak length of eight spectra) extraction. The recursive feature extraction was enabled. The ion deconvolution occurred with an Extracted ion chromatogram (EIC) correlation of 0.8 using [M + H]^+^ as primary ion, [M + Na]^+^ and [M + K]^+^ as adducts and [M + H-H_2_O] as common ion. Then a within-batch correction was employed to fix the maximal QC variance at 40 % before the correction and at 20 % after. Finally, the annotation process used an inhouse laboratory standard list (*n* = 100) based on MS pattern of pure analytical standard and used the Metlin online database (mSigma<20), the Kegg Compound Database and OligoNet database. Multivariate Statistical data Analysis (MVA) was performed using the R software (v 4.0.3). Principal Component Analysis (PCA) from the FactoMineR package was used to identify potential data separation through qualitative factors (i.e. grape variety and tannin potential). Partial Least Square Discriminant Analysis (PLS-DA) was performed using the mixOmics R package with the barrel tannin potential as discriminant variable. The model diagnostic employed the Area Under the Receiver Operating Characteristic (AUROC curve) which is particularly suitable in two group discrimination (low and medium tannin potential) metabolomic studies (Szymańska et al., 2012). Non-parametric Kruskal-Wallis tests and multiple comparisons of treatment were performed through the agricolae package and enabled post hoc test using the criterium Fisher's Least Significant Difference adjusted by the False Discovery Rate method.

## 3 Results and discussions

### 3.1 Impact of grapevine variety and oak wood tannin potential on wine's oxidative stability during bottle aging

White wines antioxidant capacity mainly relies on nitrogen and sulfur-containing compounds originated from grapes and yeast/bacteria autolysis during on-lees aging (Nikolantonaki et al., 2018; Romanet et al., 2019; Romanet et al., 2020; Romanet et al., 2021; Roullier-Gall et al., 2019). Moreover, oak wood extracted compounds (i.e. ellagitannins) are highly reactive with dissolve oxygen and in that respect they are considered as contributors to the wine native antioxidant metabolome protectors (Nikolantonaki et al., 2019). The same study put in evidence a direct positive correlation between the tannin potential and wines oxidative stability regardless of the grape variety and the vintage. However, profiles of ellagitannin extraction kinetics were dependent on the grape variety. CHA wines presented a linear kinetic profile while SAU wines presented a bell-shaped kinetic profile with a maximum observed at 4 months of barrel aging. These results indicated for the first time, that wood derived compounds other than ellagitannins also contribute to wines antioxidant capacity.

**Fig. 1:**
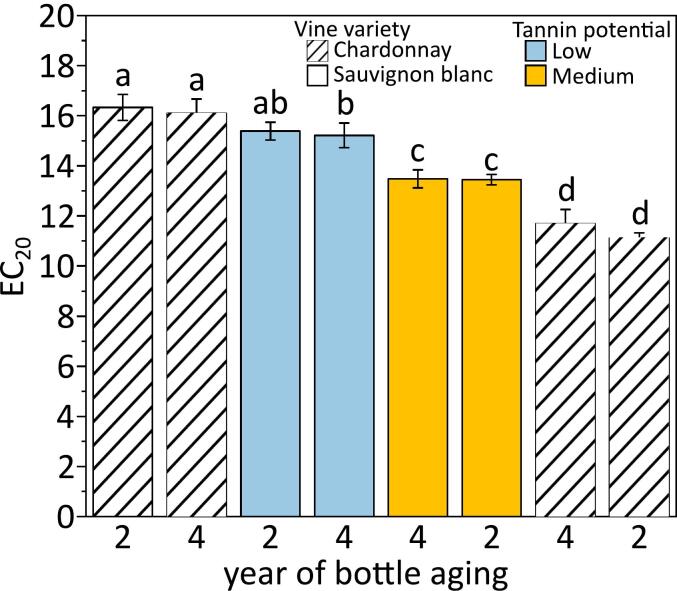
Comparison of the antioxidant capacity of Chardonnay (hatches) and Sauvignon blanc (full) wines aged in low tannin potential (blue) or in medium (yellow) TP at 2 and 4 years of bottle aging under cellar conditions. Fisher's Least Significant Difference post-hoc tests were employed to reveal statistical differences indicated through different letters (p < 0.05). (For interpretation of the references to colour in this figure legend, the reader is referred to the web version of this article.)

In order to go further in that perspective, the same wines considered in our previous study (Nikolantonaki et al., 2019) were analyzed after 2 and 4 years of bottle aging. Wines global antioxidant capacity was estimated by the DPPH method optimized for white wines assay and results were expressed as EC_20_ (Fig. 1)_._ The higher the EC_20_ value, the lower the antioxidant capacity of the wine (Romanet et al., 2019). MTP barrels triggered significantly higher antioxidant capacity to wines (EC_20_ ranked between 13.5 and 11.1) compared to those from LTP barrels (EC_20_ value set between 16.3 and 15.2). These results corroborated previous observations based on electronic paramagnetic resonance and confirmed that MTP better preserved the native wine antioxidant metabolome (Nikolantonaki et al., 2019). Differences revealed important interactions between the wine matrix and the compounds extracted from barrels with different TPs (Fig. 1). Regardless of the TP, wines global antioxidant capacity was dependent to the grape variety. Indeed, CHA wines had significatively (*p* < 0.05) greater antioxidant capacity than SAU wines, at 2 and 4 years of bottle aging.

### 3.2 Impact of the oak wood tannin potential on wine molecular composition

**Fig. 2:**
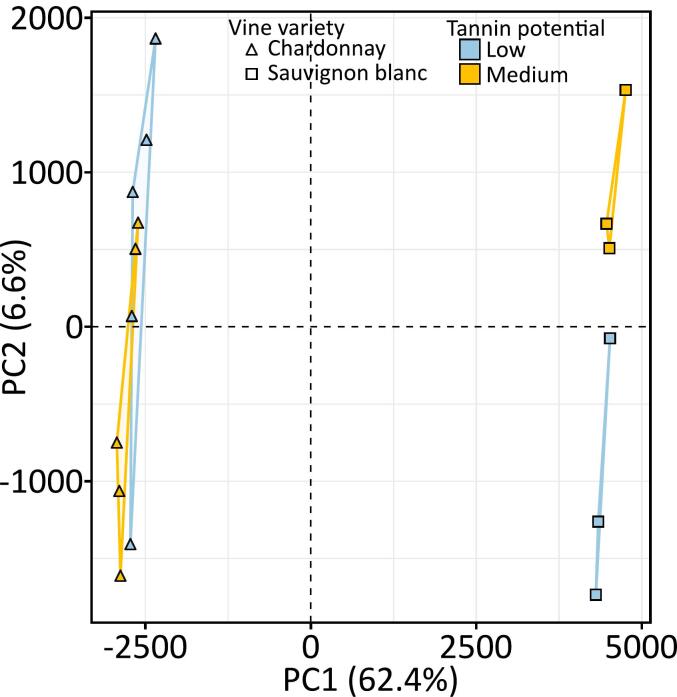
PCA score plot of untargeted UPLC-Q-ToF-MS/MS analysis in negative mode of Chardonnay (triangles) and Sauvignon blanc (squares) aged in low (blue) or medium (yellow) tannin potential after four years of bottle aging. (For interpretation of the references to colour in this figure legend, the reader is referred to the web version of this article.)

As shown recently, the absolute quantitation of known antioxidant (i.e. glutathione and glutathionylcaftaric acid, phenolic acids, flavan-3-ols and tyrosol) is not sufficient to explain the white wine oxidative stability (Nikolantonaki et al., 2019). To go further we performed a UPLC-Q-Tof-MS based non-targeted metabolomic approach, using MS-MS data dependent analysis on QCs to increase the reliability of annotations. Analysis encompassed ten bottles of CHA (5 aged in LTP and the remaining 5 aged in MTP) and six bottles of SAU (3 aged in LTP and the remaining 3 aged in MTP). We used principal component analysis (PCA) to observe similarities and dissimilarities among chemical fingerprints based on the relative abundance of a total of 1903 detected features (Fig. 2). PCA enabled remarkable and straightforward separation among grape varieties through the first axis that accounted for 62.4 % of the overall dataset variance explanation. The second axis (PC2: 6.6 %) enabled the separation of SAU samples according to their barrel TP, whereas it was not the case for CHA wines. Thus, the SAU molecular matrix appeared more impacted by the TP, even after 4 years of bottle storage, whereas the CHA matrix signature remained more conservative. These results are in accordance with winemakers' observations, which assume that CHA wines better fit with a long on-lees aging in new oak barrels.

**Fig. 3:**
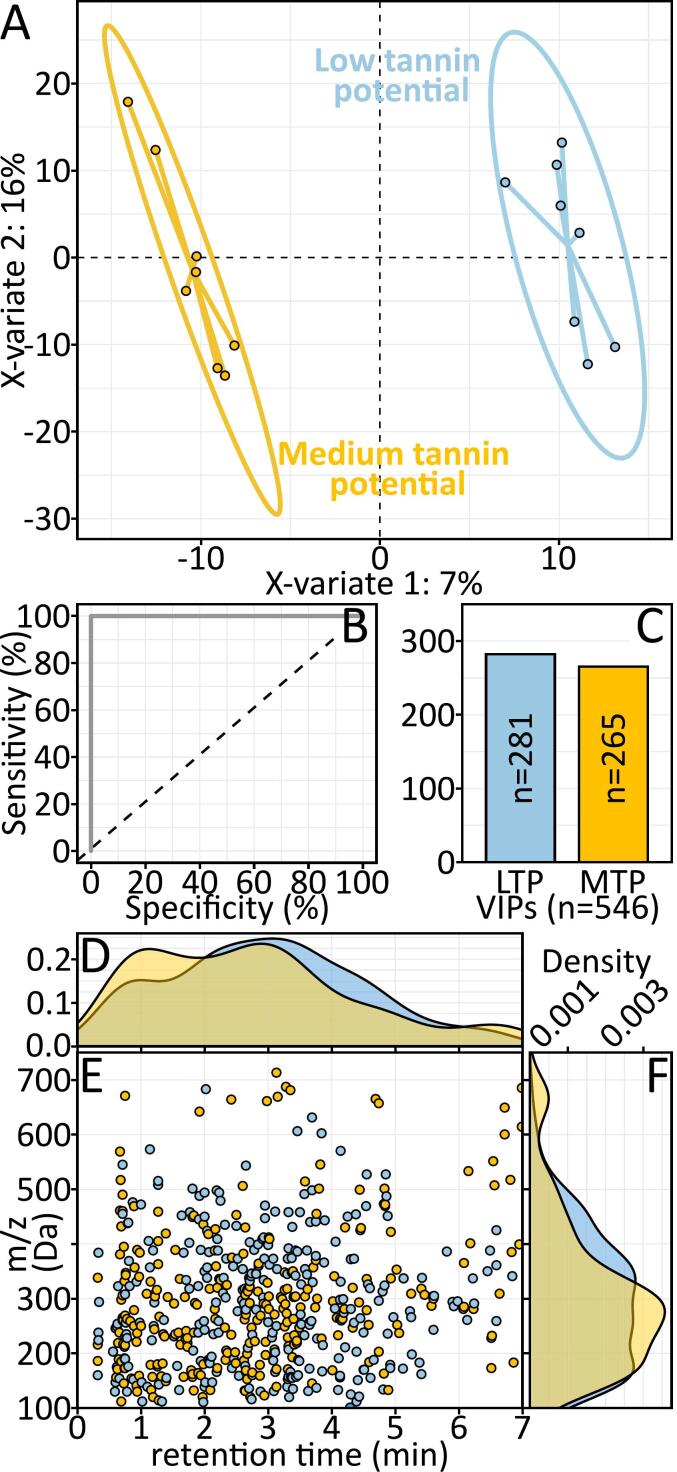
Supervised classification using partial least squares discriminant analysis (PLS-DA) of UPLC-Q-ToF-MS/MS data from all wines (CHA + SAU) after four years of bottle storage, with the tannin potential (low: blue; medium: yellow) as discriminant variable (A). The model diagnostic was evaluated through the AUROC curve (B). The relevant features (VIPs) were presented as histogram (C). A diagram was employed to present the retention time (D) and mass range (F) diversity as density plot and the scatter plot (E) summarize both information. (For interpretation of the references to colour in this figure legend, the reader is referred to the web version of this article.)

To emphasize the separation of wine samples according to barrel TP after 4 years of bottle aging, and in order to identify TP biomarkers, we performed a partial least square analysis using the barrel TP (LTP or MTP) as discriminant factor (PLS-DA). Both CHA and SAU wines samples were discriminated along the first component according to the TP, based on 1903 molecular features (Fig. 3A). The developed model enabled a perfect specificity and selectivity that ensures the wine discrimination according to the TP (Fig. 3A-B). Features relevant to that discrimination were identified using the Variable Importance in the Projection method (VIP > 1; Fig. 3C), thus it appears that among the 546 VIPs, 281 features were more specific to LTP when the 265 remaining were more specific to MTP. Fig. 3.E presents the diagram made of the VIPs represented in function of their couple retention time (rt) and mass to charge (*m/z*) ratios. The distribution of the LTP linked VIPs revealed a higher density in medium retention time (about 3 min) and covered a wide mass range from 100 to 500 Da. The MTP linked VIPs exhibited a bimodal distribution with a first maxima set at 1 min and a second at 3 min and covered a more restricted mass range (100–300 Da).

Finally, the annotation process was based on an inhouse laboratory standard list (*n* = 100) of MS patterns of pure analytical standard compounds, the analyte list (Knapsack and Phenol Explorer) and spectral library matches (Fiehn Natural Product, Mass Bank). Among the 281 LTP VIPs, 88 presented putative annotations and 29 belonged to amino acid derivatives. We identified 4 amino-acids (L-Arginine; l-Glutamine; L-Phenylalanine; L-Aspartic Acid), 5 dipeptides (Trp Pro-OH; Trp Pro; Val Trp-OH; Ala Pro; Phe Asp), 10 tripeptides (Ile Pro Lys; Ser Val Leu; Met Glu Gly; Ser Gly Phe; Leu Leu Ala; Thr Ala Phe; Leu Leu Lys; Pro Leu Ile; Gln Lys Pro; Val His Ala), 9 tetrapeptides (Ala Ala Ser Val; Ile Gly Ala Ile; Ala Ala Leu Val; Ala Asn Pro Ser; Asp Lys Lys Thr; Ala Asn Pro Ser; Gly Asn Ser Tyr; Ala His Ile Thr; Ala Lys Val Ser) and 1 pentapeptide (Ala Val Val Pro Leu). MTP aged wines also revealed the importance of amino acid derivatives with 27/66 putative annotations that encompassed 4 amino acids (L-Leucine; L-Tryptophan; L-Tyrosine; 5-Hydroxy-L-tryptophan), 15 dipeptides (Phe(4-Cl) Ser-OH; Gly Leu; Ser Tyr; His Phe4Cl-OH; Phe Thr; Trp Ser; Val Val-OH; Gly Trp; Trp Ala; Trp Ser; Val Trp; Ile Trp; Val Trp; Tyr Val; Ser Pro), 4 tripeptides (Ala Ile His; Pro Ser Thr; Ala Glu Pro; Ala Asp Ile) and 4 tetrapeptides (Asp His Pro Thr; Ala Ala Thr Thr; Phe Ile Ile Asn; Gly Pro Pro Ser). The antioxidant dipeptides characteristics of MTP aged wines appeared enriched in tyrosine or tryptophan residues (9/14). Zheng et al. (2016) proved that Tyr, Trp, Cys and Met residues had the ability through electron/hydrogen transfer to scavenge radicals. Moreover, annotations also revealed higher content in gallic acid (*m*/*z* 171.0286, rt. 3.31 min) in wines aged in MTP that might contribute to their higher antioxidant capacity compared to wines aged in LTP (cf Figure. 1; Roussis & Sergianitis, 2008).

### 3.3 Impact of the oak wood tannin potential on the targeted volatile profiles

**Fig. 4:**
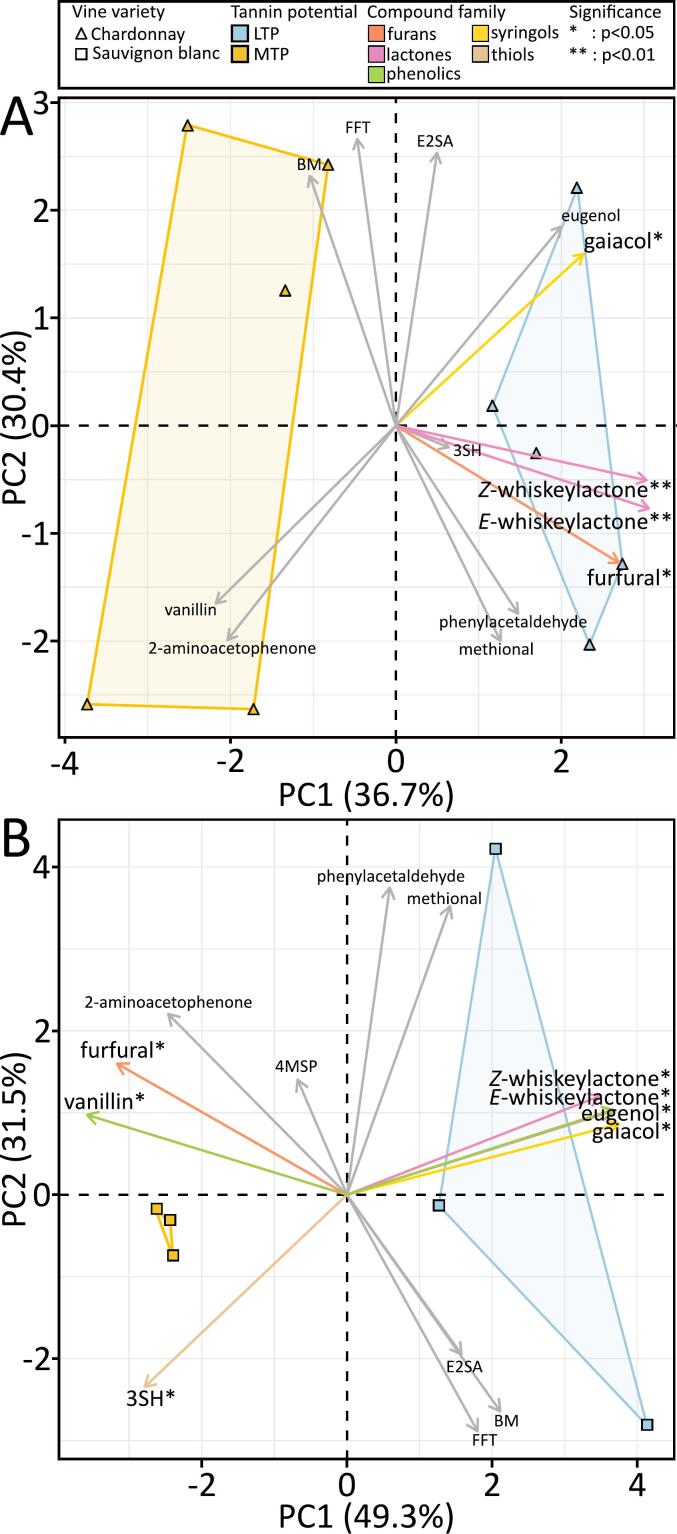
PCA biplots of the first two PCs for the absolute quantitation of the 11 volatile compounds analyzed for both Chardonnay (triangles, A) and Sauvignon blanc (squares, B) wines aged in low tannin potential (blue) or in medium TP (yellow) after four years of bottle aging. Significant different amount in compound were symbolized by “*” for *p* < 0.05 and by “**” for *p* < 0.01 following compound name. (For interpretation of the references to colour in this figure legend, the reader is referred to the web version of this article.)

The wine volatile profile is determined by the vine variety that influenced both physico-chemical properties and aroma compounds (Karabagias et al., 2020) and might be affected by wine oxidative status (Mayr et al., 2015; Pinto et al., 2018). Barrel aging is considered to enrich the wine in volatile aroma depending on the wood variety and species, the tannin potential (Díaz-Maroto et al., 2008) and the toasting processes (Vichi et al., 2007). Fig. 4 shows the PCA analysis of GC–MS/MS data from CHA and SAU wines after 4 years of bottle storage, and Table 1 gathers the corresponding measured concentrations. The analysis included SAU varietal thiols (4MSP: 4-methyl-4-sulfanylpentan-2-one, E2SA: ethyl-2-sulfanylacetate, 3SHA: 3-sulfanylhexyl acetate, 3SH: 3-sulfanylhexan-1-ol, 2-furfurylthiol (FFT) and benzenemethanethiol (BM). 3SHA was detected but remained under the quantitation limit (<LOQ: Table 1). Six oak derived compounds associated either with the wood (*E* and *Z*-whiskeylactones) or with the toasting (furfural, gaiacol, eugenol and vanillin) were considered (Díaz-Maroto et al., 2004; Koussissi et al., 2009). Methional, 2-phenylacetaldehyde and 2-aminoacetophenone concentrations, related to wines oxidation off-flavors (Hoenicke et al., 2002; Mayr et al., 2015) were not discriminant for a given TP, for both CHA and SAU wines. Moreover, these concentrations remained very low even after four years of bottle aging, with values in the lower range of those commonly found in wines, and associated with young or fresh wines (Culleré et al., 2007; Schmarr et al., 2007).

**Table 1:** Average concentrations of the 15 volatiles compounds quantified in Chardonnay (n= 5 replicates) and Sauvignon blanc (n= 3 replicates) wines aged in low and medium tannin potential barrels. For each compound, superscripts a and b indicate significantly different values (Kruskall-Wallis p-value significance: *<0.05, **<0.01, ***<0.001.

Compound		Chardonnay				Sauvignon blanc			
Assignment	Family	X^2^	p	LTP	MTP	X^2^	p	LTP	MTP
14MSP	thiols			<LOQ	<LOQ			4.16 ± 3.32	5.37 ± 0.506
1E2SA	thiols	0.01		1360±199	1350±162	0.05		1020±145	998 ± 62.5
1FFT	thiols	0.88		11.5 ± 2.6	14 ± 4.5	0.05		19.87 ± 5.26	18.1 ± 2.54
1BM	thiols	1.32		14.7 ± 2.3	17.2 ± 3.1	0.43		14.66 ± 2.06	13.5 ± 1.48
13SHA	thiols			<LOQ	<LOQ			<LOQ	<LOQ
13SH	thiols	0.27		867±137	886±175	3.86	*	452.4 ± 88.1^b^	641 ± 57.7
2methional	aldehydes	2.45		1.06 ± 0.22	0.78 ± 0.55	1.19		1.05 ± 0.54	0.682 ± 0.0531
2phenylacetaldehyde	aldehydes	0.53		8.04 ± 1.22	7.23 ± 1.32	0.05		5.43 ± 0.91	5.07 ± 0.189
22-aminoacetophenone	ketones	1.32		0.058 ± 0.011	0.069 ± 0.016	2.33		0.086 ± 0.012	0.0975 ± 0.007
2furfural	furans	4.81	*	2370±413	1690 ± 157^b^	3.86	*	960 ± 656^b^	1530 ± 97.2
2gaiacol	syringols	4.81	*	3.39 ± 0.17	3.05 ± 0.31^b^	3.86	*	2.66 ± 0.12	1.93 ± 0.0328^b^
2*E*-whiskylactone	lactones	6.82	**	353±21	227 ± 22^b^	3.86	*	654 ± 35.8	402 ± 17.9^b^
2*Z*-whiskylactone	lactones	6.82	**	906±45	538 ± 32^b^	3.86	*	710 ± 37.8	595 ± 33.3^b^
2eugenol	phenolics	3.15		15.3 ± 0.7	13.8 ± 1.7	3.86	*	21.7 ± 1.13	11.9 ± 0.74^b^
2vanillin	phenolics	3.15		124±6	141±13	3.86	*	87.6 ± 11.7^b^	110 ± 6.12

1 Compounds are expressed in ng.L^−1^.

2 Compounds are expressed in μg.L^−1^.

The PCA presented as biplot was based on the absolute quantitation of the 13–14 compounds mentioned above and explained respectively 67.1 % and 80.8 % of the overall variability for CHA (Fig. 4A) and SAU (Fig. 4B) samples. The two wine matrices were separated according to their barrel TPs through the first PC, mostly according to oak-related aromas (Fig. 4). Furfural, *E* and *Z*-whiskey-lactones were the most significant contributors to the separation between LTP and MTP for CHA wines (Fig. 4A). The difference between SAU LTP and MTP was driven by furfural, gaiacol, *E*-whiskeylactone, *Z*- whiskeylactone, eugenol and vanillin (Fig. 4B). These observations were strengthened through Kruskal-Wallis one-way analysis of variance tests performed on the absolute metabolite quantitation (Table. 1). Higher concentrations of the native wood aroma whiskylactones in wines aged in LTP barrels is consistent with the analysis of a large series of French oaks, which showed that woods with low tannin concentrations (more often from the sessile species, on average) were significantly richer in whiskylactones (Feuillat et al., 2003). However, our results further confirm that the combined impact of wood toasting and wood tannin potential on the volatile composition (and related sensory attributes) of wines is matrix dependent, since the heat-related furfural and vanillin compounds are anti-correlated for CHA wines, and correlated for SAU wines (Fig. 4, Table 1).

## 4 Conclusion

The present study provided an overview of the oak wood tannin potential influence on white wines antioxidant stability and on their related chemical signatures. Barrels made of medium tannin potential staves strengthen the wine antioxidant capacity of wines during up to four years of bottle aging. Untargeted high-resolution mass spectrometry coupled to unsupervised multivariate statistical analysis could clearly discriminate wines according to the grape variety after four years of bottle aging. Sauvignon blanc wines further appeared to be separated according to the tannin potential, in contrast to Chardonnay wines, suggesting that the native metabolome of the latter was less affected by barrel aging. Supervised (PLS-DA) analysis of all wines after bottle aging highlighted the extensive oak wood contribution (546 VIPs) to the wine chemical fingerprints (1903 features) regardless of the grape variety. Medium tannin potential barrels were associated with higher concentrations in dipeptides containing antioxidant residues as well as in known antioxidant biomarkers such as gallic acid. Barrels with low tannin potential were characterized by higher concentrations in whiskylactones, while wines aged in barrels with medium tannin potential exhibited higher concentration in vanillin.

## CRediT authorship contribution statement

**Kevin Billet:** Writing – review & editing, Writing – original draft, Visualization, Validation, Formal analysis, Data curation. **Cécile Thibon:** Writing – review & editing, Formal analysis. **Marie Laure Badet:** Writing – review & editing, Resources, Investigation, Funding acquisition. **Nolwenn Wirgot:** Formal analysis, Data curation. **Laurence Noret:** Formal analysis. **Maria Nikolantonaki:** Writing – review & editing, Validation, Supervision, Methodology, Investigation, Funding acquisition, Conceptualization. **Regis D. Gougeon:** Writing – review & editing, Validation, Supervision, Project administration, Methodology, Investigation, Funding acquisition, Conceptualization.

## Declaration of competing interest

The authors declare that they have no known competing financial interests or personal relationships that could have appeared to influence the work reported in this paper.

## Data Availability

Data will be made available on request.

## Appendix A Supplementary data

Supplementary Table 1. Experimental set-up of Chardonnay and Sauvignon white wines analyzed in biological replicates (CHA/2y: n= 6, CHA/4y: n= 10; SAU/2y: n= 4, SAU/4y: n= 6) at 2 (2y) and 4 (4y) years of bottle aging. Image 1
