# Supplementary material for: White wines aged in barrels with controlled tannin potential exhibit correlated long-term oxidative stability in bottle
Source: Food Chem X. 2024 Oct 16;24:101907. doi: 10.1016/j.fochx.2024.101907 (PMC11547895; doi:10.1016/j.fochx.2024.101907)
Supplement: Supplementary file 1 — Supplementary Table 1. Experimental set-up of Chardonnay and Sauvignon white wines analyzed in biological replicates (CHA/2y: n= 6, CHA/4y: n= 10; SAU/2y: n= 4, SAU/4y: n= 6) at 2 (2y) and 4 (4y) years of bottle aging. [file mmc1.docx]

**White wines aged in barrels with controlled tannin potential exhibit correlated long-term oxidative stability in bottle**

Kevin Billet^a,1^, Cécile Thibon^b^, Marie Laure Badet^c^, Nolwenn Wirgot^a^, Laurence Noret^a^, Maria Nikolantonaki^a^, Regis D. Gougeon^a*^

**Supplementary materials**

Supplementary Table 1. Experimental set up of Chardonnay and Sauvignon blanc white wines analyzed in biological replicates (CHA/2y: n=6, CHA/4y: n=10; SAU/2y: n=4 SAU/4y: n=6) at 2 (2y) and 4 (4y) years of bottle aging.

| Vine variety | Barrel tannin potential | Bottles | | Analysis | | |
| --- | --- | --- | --- | --- | --- | --- |
|  |  | Storage  (year) | Number  (n) | DPPH | LC-Q-Tof-MS | GC-/MSMS |
| Chardonnay | LTP | 2 | 3 | X |  |  |
|  |  | 4 | 5 | X | X | X |
|  | MTP | 2 | 3 | X |  |  |
|  |  | 4 | 5 | X | X | X |
| Sauvignon blanc | LTP | 2 | 2 | X |  |  |
|  |  | 4 | 3 | X | X | X |
|  | MTP | 2 | 2 | X |  |  |
|  |  | 4 | 3 | X | X | X |

 
 
